# An unexpected advantage of insectivorism: insect moulting hormones ingested by song birds affect their ticks

**DOI:** 10.1038/srep23390

**Published:** 2016-03-21

**Authors:** Sándor Hornok, Dávid Kováts, Barbara Flaisz, Tibor Csörgő, Árpád Könczöl, György Tibor Balogh, Attila Csorba, Attila Hunyadi

**Affiliations:** 1 Department of Parasitology and Zoology, Faculty of Veterinary Science, Szent István University, 1078 Budapest, Hungary; 2 Department of Evolutionary Zoology and Human Biology, University of Debrecen, 4032 Debrecen, Hungary; 3 Ócsa Bird Ringing Station, 2364 Ócsa, Hungary; 4 Department of Anatomy, Cell- and Developmental Biology, Eötvös Loránt University, 1117 Budapest, Hungary; 5 Compound Profiling Laboratory, Gedeon Richter Plc., 1103 Budapest, Hungary; 6 Institute of Pharmacognosy, University of Szeged, 6720 Szeged, Hungary

## Abstract

Ecdysteroids are important hormones that regulate moulting in arthropods. Three-host ixodid ticks normally moult to the next stage after finishing their blood meal, in the off-host environment. Presumably, three-host ticks that feed on the blood of insectivorous vertebrate hosts can be exposed to high levels of exogenous ecdysteroids causing them to initiate apolysis (the first step of moulting) on the vertebrate host. The aim of the present study was to investigate whether ticks undergo apolysis on insectivorous song birds, and if this phenomenon is associated with the seasonal variation in the availability of moths and with the presence of naturally acquired ecdysteroids in avian blood. During a triannual survey, 3330 hard tick larvae and nymphs were collected from 1164 insectivorous song birds of 46 species. A noteworthy proportion of ticks, 20.5%, showed apolysis. The occurrence of apolytic ticks on birds was correlated with the known seasonality of lepidopteran caterpillars. In addition, 18 blood samples of tick-infested birds were analysed with liquid chromatography – tandem mass spectrometry. Eight samples contained ecdysteroids or their derivatives, frequently in high concentrations, and the presence of these was associated with tick apolysis. In conclusion, naturally acquired ecdysteroids may reach high levels in the blood of insectivorous passerine birds, and will affect ticks (feeding on such blood) by shortening their parasitism.

Birds exhibit the most diverse range of ecological functions among vertebrates, because they participate in seed dispersal, pollination, pest control (by consuming insect parasites), carcass and waste disposal^1^. At the same time, birds are also known to play a significant role in the epidemiology of infectious diseases, *e.g.* by short- and long-distance dissemination of ixodid ticks and tick-borne pathogens^2^,^3^. The latter include several important zoonotic disease agents, such as *Borrelia burgdorferi* sensu lato that causes Lyme disease^4^, *Anaplasma phagocytophilum* responsible for granulocytic anaplasmosis^5^, and tick-borne encephalitis virus^6^. Although frequently studied separately, these two facets of avian life are necessarily interrelated, *i.e.* the niche birds occupy in an ecosystem will influence the risk they may pose as a source of ticks and tick-borne pathogens towards humans and their domestic animals.

In this host-parasite relationship, ticks may affect birds in several ways. For instance, infestation with certain tick species may cause disease in avian hosts^7^,^8^. Ticks may also inoculate tick-borne pathogens into birds, with or without pathological consequences^4^,^5^,^9^,^10^. On the other hand, the species-specific characteristics of birds will influence their tick infestation. The feeding level of birds (ground vs. arboreal) will have a significant impact on their tick burden, depending on the questing height different tick species occupy on the vegetation^10^,^11^. In addition, bird species may differ in how they interact with their ixodid ticks at the site of the tick bite. While ticks are known to inject salivary components to promote their prolonged blood feeding, the host’s immune system also mounts a local immune response against the tick^12^.

In the temperate climate zone, birds usually harbour larvae and nymphs of three-host ixodid ticks^3^. After blood feeding, these immature stages fall from their host, and moult to the next stage in the off-host environment. The moulting process is under control of moulting hormones, the so-called ecdysteroids^13^. However, on-host initiated moulting, *i.e.* apolysis (detachment of the previous cuticle) of three-host ticks has recently been reported in ticks feeding on goats and bats^14^,^15^. A plausible explanation for this phenomenon was that goats may ingest phytoecdysteroids with the plants in their forage^14^, whereas bats feed on insects that may contain ecdysteroids.

To evaluate this phenomenon further in the present study, ticks collected from insectivorous song birds (Aves: Passeriformes) during a tri-annual survey were examined for signs of apolysis, and blood samples of tick-infested birds were analysed for the presence of ecdysteroid moulting hormones. In this context, passerine birds appear to be particularly suitable subjects to study, because caterpillars constitute a significant portion of their diet (especially during the nesting period)^16^, and caterpillars are known for their high ecdysteroid concentrations^17^.

## Results

During the three-year period, a total of 3330 ixodid ticks were collected from 1164 passerine birds (representatives of 46 mainly or partly insectivorous species). Larval and nymphal ticks of *Ixodes* spp. predominated, followed by *Haemaphysalis concinna*, accounting for 70.3% (2341 out of 3330, CI: 68.7–71.9%) and 29.7% (989 out of 3330, CI: 28.2–31.3%) of all collected ticks, respectively.

**Figure 1:**
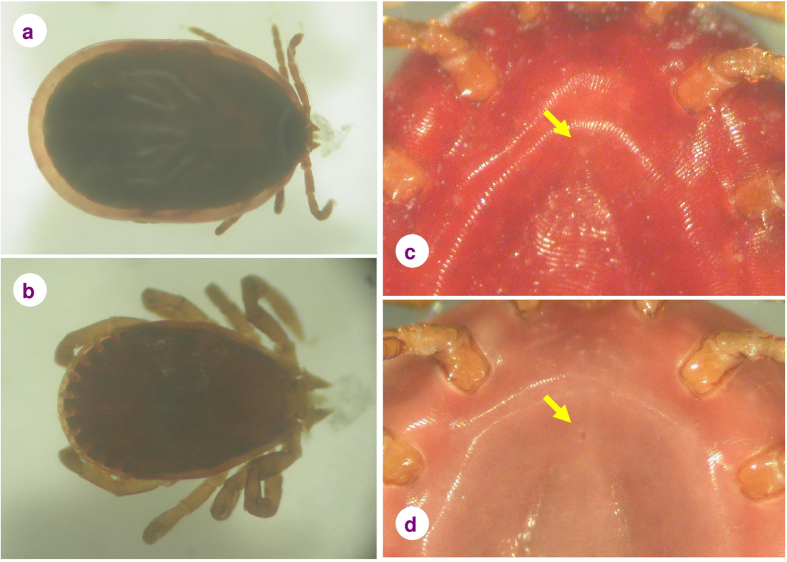
*Haemaphysalis concinna* nymphs showing apolysis: (**a**) when close to full engorgement, (**b**) at the beginning of engorgement. Compared to nymphs that did not show the signs of apolysis (**c**), the place of the genital pore (arrow) was more apparent on apolytic nymphs (**d**).

**Figure 2:**
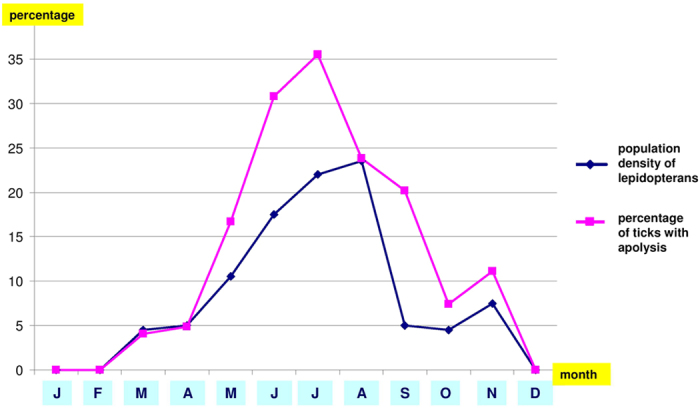
The monthly regional population density of lepidopterans and the percentage of ticks showing apolysis. The latter indicates the number of apolytic ticks expressed as the percentage of all ticks removed from birds, calculated for each month.

A noteworthy proportion, 20.5% (683 out of 3330, CI: 19.2–21.9%) of tick larvae and nymphs collected from birds showed apolysis, the initiative act of moulting (Fig. 1a). In the case of engorged apolytic nymphs, the place of the genital pore (which will open only in the adult stage) was frequently darker and more visible (Fig. 1c,d). The signs of apolysis were also observed in the case of unengorged ticks, *i.e.* at the beginning of their blood meal (Fig. 1b). The percentage of apolytic ticks on birds was the highest in July (35.5% = 250/705, CI: 31.9–39.1%). The monthly proportion of apolytic larvae and nymphs was highly associated with the reported regional monthly population density of lepidopterans (Spearman’s rank correlation: r = 0.93, P = 0.00001) (Fig. 2).

**Figure 3:**
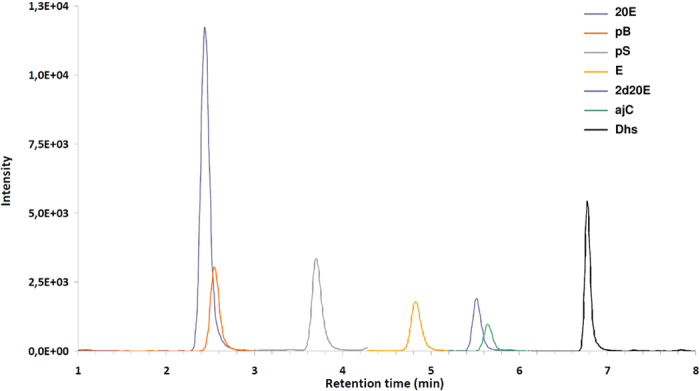
MRM chromatogram of sample No. 6. Abbreviations can be found in the legend of Table 2, sample data are shown in Table 1.

**Figure 4:**
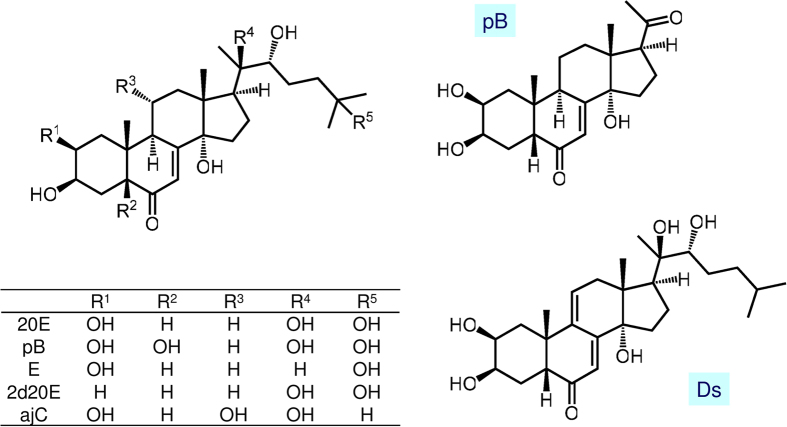
Structures of the ecdysteroids tested in the blood samples.

Blood samples of 18 tick-infested birds were tested for the presence of ecdysteroids. In eight samples, up to seven ecdysteroids or their derivatives were present in detectable quantities (Fig. 3; Table 1). Structures of the ecdysteroids tested in the blood samples are summarized in Fig. 4. The proportion of ecdysteroid-positive samples was higher in the summer (61.5% = 8/13) than the spring (0.0% = 0/5) and this difference was statistically significant (χ2 = 5.539, df = 1, P = 0.019). The proportion of ecdysteroid-positive samples in birds with apolytic ticks (87.5% = 7/8) was almost nine times higher than in birds with no apolytic ticks (10.0% = 1/10) and this difference was statistically significant (χ2 = 10.811, df = 1, P = 0.001).

## Discussion

Moulting hormones (ecdysteroids) have a similar role (*i.e.* triggering and controlling moulting) in ticks as in other arthropod groups^13^. It was observed that when ecdysteroids are produced in tick tissues, their levels rose slowly during the seven days of blood feeding and during the first ten days after detachment from the host^13^. During this period, ecdysteroids reached a concentration of only about 50 ng/ml haemolymph. However, this was followed by a sharp rise of ecdysteroid level, which peaked at 500 ng/ml at ~15 days after drop off from the vertebrate host^13^. As the first step of moulting, apolysis of ticks is induced by (and occurs concomitantly with) such highly elevated ecdysteroid titers^13^. Thus in nature, moulting of three-host ticks occurs exclusively in the off-host environment. On the other hand, when ticks are provided with ecdysteroid-containing blood (*i.e.* an exogenous source of moulting hormones), it will accelerate their moulting and will induce apolysis in a dose-dependent way^18^. Investigation of the natural occurrence of the latter phenomenon, to the best of our knowledge, has never been reported.

In the present survey, the percentage of ticks showing apolysis on birds was the highest in July, following the regional peak activity of caterpillars (May-June)^16^. Caterpillars predominate in the food of forest-dwelling passerine birds^19^, and their proportion can exceed 90% in the diet of nestlings^16^. Caterpillars are also known to contain high titres of ecdysteroids, up to 780 ng/g^17^. Throughout the study, a highly significant seasonal correlation was demonstrated between the percentage of apolytic ticks on birds, and the monthly population density of lepidopterans. This association can be interpreted in light of one common “preceding factor”, *i.e.* the presence of ecdysteroids in both the caterpillars and the blood of the birds. In other words, both the emergence of adult lepidopterans and the apolysis of ticks feeding on insectivorous birds can be regarded as a consequence of moulting hormones in caterpillars. The level of ecdysteroids in caterpillars effecting metamorphosis is likely to have similar physiological effects on immature ticks, based on the universal signal (analogous functions) they represent among arthropods (in general) and in ticks^20^. However, we recognize that the results from the present study are all correlative in nature and further experimental work is therefore required to establish a causal relationship between insect-derived ecdysteroids in the avian diet and on-host apolysis of hard ticks.

A second, lower peak of bird tick apolysis was observed in the month of November following its steady decline from the summer peak. One possible explanation for the November peak is that several song bird species switch from fully insectivorous to partly frugivorous and/or granivorous diet during the autumn, when insects are less available^21^. Relevant fruits (*e.g.* various berries) typically ripen from August in Central-Eastern Europe, and some (*e.g. Malus* spp.) were reported as ecdysteroid-positive by means of radioimmunoassay^22^.

Prior to apparent apolysis, untimely ingestion of large amounts of exogenous ecdysteroids may have detrimental consequences for ticks, because these hormones are known to have an antifeedant effect and to induce (earlier) salivary gland degeneration^18^,^23^, thus shortening the duration of blood feeding. These effects are particularly relevant in the case of ticks exposed to high levels of ecdysteroids at the beginning of their blood meal, as suggested by unengorged ticks showing apolysis in the present study. As the duration of blood feeding after tick attachment increases the risk of transmission of tick-borne pathogens^24^,^25^, the present findings may have epidemiological implications and are relevant for designing future strategies to control tick infestations and the risk of tick-borne diseases.

A large-scale feeding assay on newly-hatched Japanese quails found that foods containing high levels of ecdysteroids, such as Leuzea seeds, exerted anabolic activity and had beneficial effects on the birds^26^. A subsequent study with pure 20-hydroxyecdysone (20E) could also connect this dose-dependent anabolic activity in the birds to the ecdysteroid content of the diet^27^. In the group of birds with *ad libitum* access to the seeds during the 50-day experiment, the 20E levels reached a concentration of 80 ng/ml in the blood serum (as measured by a radioimmunoassay). In addition, the levels of 20E in the blood serum of the birds were proportional to the amount of seeds consumed^26^. This result indicates that the metabolism and/or elimination of ecdysteroids cannot be as rapid in birds as previously described for rodents (8.15 min half-life of 20E in mice)^28^, and that the accumulation of these compounds in birds is in fact possible. Our results on insectivorous wild passerine birds showed a much greater accumulation of 20E as well as of ecdysone in several individuals, reaching very high levels of up to ca. 8 μg/ml and 2 μg/ml of these two compounds, respectively.

In addition to this result, the presence of five other ecdysteroids as well as their unexpectedly high levels within several blood samples raise a number of questions concerning their origin and the mechanism of accumulation. These compounds occur in plant species belonging to different families including the Asteraceae (*e.g.* Serratula, Leuzea), Lamiaceae (*e.g.* Ajuga), and Caryophyllaceae (*e.g.* Silene)^29^, but the concentration of these compounds is typically an order of magnitude lower than that of 20E. Among them, the presence of poststerone and 2-deoxy-20-hydroxyecdysone might have a straightforward possible explanation. Poststerone has previously been identified as a major *in vivo* metabolite of 20E in mice^30^. Moreover, the 20,22 side-chain cleavage of cholesterol, initiated by the build-up of the corresponding vicinal diol, is the first step in the biosynthesis of the mammalian steroid hormone, which is catalysed by the enzyme cytochrome P450 11A1 (CYP11A1)^31^. Considering that the machinery for steroidogenesis appears to be highly conserved throughout the entire animal kingdom^32^, such a transformation of the 20,22-diol containing steroid 20E is also likely to occur in birds. However, as 2-deoxy-20-hydroxyecdysone was identified as a metabolite in human urine after consuming 20E^33^, it cannot be excluded that this compound was also present in the blood of the birds as a metabolite of dietary 20E.

On the other hand, the significant amounts of polypodine B, ajugasterone C, and particularly dacryhainansterone in the blood samples is very surprising: their structure makes it unlikely that they are the metabolic products of 20E in the diet of the birds. We therefore assume that these phytoecdysteroids made their way from plant sources through caterpillars to the birds, and, eventually, into the ticks. Amazingly, these compounds were detected in amounts comparable to or even higher than that of 20E, which strongly suggests that their metabolism and/or elimination is much slower. Based on the few available studies on the metabolism of ecdysone in mice^34^,^35^ and of 20E in rodents^30^,^36^ and in humans^33^,^37^, reduction at the B-ring is among the major metabolic routes of ecdysteroids. Moieties like a 5α-OH forming intramolecular H-bond with the 6-oxo group (polypodine B) or a conjugated 7(9,11)-dien-6-one (dacryhainansterone) might interfere with this process, and the lack of OH-25 (ajugasterone C and dacryhainansterone) can possibly decrease phase II metabolism *i.e.* sulphate or glucuronide conjugation. It should also be noted, that all these compounds, and mainly dacryhainansterone, are more lipophilic than 20E, based on which other pharmacokinetic properties (absorption, plasma protein binding *etc*.) can also significantly contribute to a relatively higher accumulation rate. To the best of our knowledge, no related studies are available with ecdysteroids other than 20E and ecdysone. Nevertheless, it is clear that the biological importance of the minor phytoecdysteroids is much greater than previously thought.

In summary, this is the first report on the presence of naturally acquired arthropod moulting hormones (ecdysteroids) in the blood of insectivorous passerine birds. Based on these results, exogenous ecdysteroids affected bird ticks by inducing on-host apolysis, which does not normally take place in the case of three-host ticks. On-host apolysis would shorten the average duration of the tick blood meal suggesting that an insectivorous diet protects birds from the full negative cost of tick feeding.

## Methods

### Sample collection

During a three year period (from January, 2012 until December, 2014), ixodid ticks were collected from passerine birds at three ringing stations in Hungary (Ócsa: 47° 17′ 54.3″ N, 19° 13′ 52.1″ E; Fenékpuszta: 46° 42′ 31.7″ N, 17° 14′ 33.8″ E; Bódva-völgy: 48° 17′ 36.3″ N, 20° 44′ 18.8″ E). Birds were mist-netted using standard Ecotone mist-nets (Gdynia, Poland), 12 m in length, 2.5 m in height and with a mesh diameter of 16 mm. The whole body of each captured bird was scrutinized for the presence of ticks. All ticks were removed with fine forceps, and put into 70% ethanol in separate vials according to their hosts. Tick species were determined according to standard keys^38^, and were consequently stored at room temperature.

In 2014, blood samples were taken from the brachial vein of some of the tick-infested birds using a fine (28G) needle and a 0.5 ml syringe (Kendall Monoject: Tyco Healthcare Group Lp., Mansfield, MA, USA). Blood samples were collected into EDTA-containing microtubes and stored frozen at −20 °C. Eighteen blood samples (from eight birds with ticks showing apolysis, and from ten birds with ticks not showing apolysis) were randomly selected for the analysis of ecdysteroids with liquid chromatography-coupled tandem mass spectrometry (LC-MS/MS) as described below.

### Sample preparation

A volume of 100 μl or 250 μl of physiological saline solution was added to the frozen blood samples. After careful homogenization, each sample was transferred to Eppendorf tubes with a Hamilton syringe. The difference between the total volume, read from the syringe, and that of the volume added was considered as the original volume of blood. Following this step, the same volume of methanol was added, the solution was homogenized by shaking and left at room temperature for at least half an hour. The precipitate was subsequently centrifuged at 10,000 rpm for 10 min at 8 °C, and the clear supernatant was utilized for LC-MS/MS studies.

### Calibration

Standard ecdysteroids 20-hydroxyecdysone (20E), poststerone (pS), ecdysone (E), 2-deoxy-20-hydroxyecdysone (2d20E), ajugasterone C (ajC) and dacryhainansterone (Ds) were obtained from previous phytochemical studies^39^,^40^, and possessed a purity of >95%. Standard stock solutions of each ecdysteroid were prepared in methanol at 1.0 mg/ml and stored at 4 °C before use. Equal volumes of the stock solutions were mixed and the obtained mixture (142.8 μg/ml for each analyte) was diluted first 100-fold and then 4-fold in serial with methanol to obtain 8 concentration levels for calibration (1428.60; 357.14; 89.29; 22.32; 5.58; 1.40; 0.35 and 0.09 ng/ml, respectively). Each calibration curve was constructed from at least six appropriate concentrations in triplicate. The limit of detection (LOD) and the limit of quantification (LOQ) were determined at the signal-to-noise ratio of about 3 and 10, respectively (Table 2).

### LC-MS/MS analysis

Experiments were carried out on an Agilent 1200 liquid chromatography system equipped with a vacuum degasser, a binary pump, an autosampler, a column temperature controller and a diode array detector. Chromatographic analysis was performed at 40 °C on a Kinetex XB-C18 column (100 × 2.1 mm, 2.6 μm) (Phenomenex, Torrance, CA, USA), with a mobile phase flow rate of 0.5 ml/min. The optimum separation was obtained under gradient elution with two isocratic time segments using 0.1% (v/v) formic acid in water as solvent A and 0.1% (v/v) formic acid in pure acetonitrile as solvent B. The linear gradient profile was: 0–0.5 min, 12% B; 0.5–2.0 min, 12–20% B; 2–3 min, 20% B; 3–9 min, 20–90% B. Post time was 6.0 min. The injection volume was set to 25 μL and the needle was rinsed and washed 3 times with methanol between injections in order to minimize carryover.

Mass spectrometry detection was performed using a 6410A triple quadrupole MS (Agilent Technologies, Palo Alto, CA, USA) equipped with an electrospray ionization (ESI) source used in positive ionization mode. The source settings were as follows: drying gas temperature, 350 °C; gas flow rate, 11 L/min; nebulizer, 40 psig; capillary voltage, 4000 V. Analyte detection was performed by multiple reaction monitoring (MRM) using an electron multiplier voltage (EMV) of 700 volts. Fragmentor voltage and collision energy (CE) were optimized individually for each target compound and are listed in Table 3. MassHunter B.04.01 was used for data acquisition and for qualitative analysis.

### Ethical approval

The study was carried out according to the national animal welfare regulations of Hungary (28/1998). Bird ringing was approved by the National Inspectorate for Environment and Nature (under licence number 14/3858-9/2012).

### Statistical analyses

Exact confidence intervals (CI) for the percentage abundances were calculated at the level of 95%. The monthly regional population density of lepidopterans was obtained from the mean monthly number of moths (Insecta: Lepidoptera), and was expressed as a percentage of the total yearly number. These data are based on the records of the Hungarian Plant Protection and Forestry Light Trap Network that were collected between 1974 and 2006, as reported^41^. Spearman rank correlation was used to test the association between the monthly proportion of apolytic ticks and the population density of lepidopterans. The association of blood ecdysteroids with season and tick apolysis (Table 1) was compared by using Chi-square test, and the differences were considered significant if P < 0.05.

## Additional Information

**How to cite this article**: Hornok, S. *et al.* An unexpected advantage of insectivorism: insect moulting hormones ingested by song birds affect their ticks. *Sci. Rep.* **6**, 23390; doi: 10.1038/srep23390 (2016).

## Figures and Tables

**Table 1:**
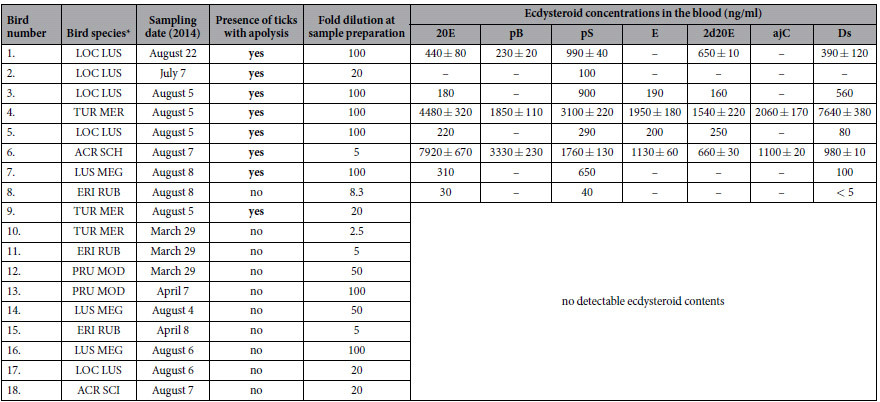
Data of eighteen tick-infested birds: the presence/absence of ticks showing apolysis and ecdysteroid concentrations in corresponding blood samples.

Abbreviations of compounds can be found in the legend of Table 2; “<” symbol denotes detectable ecdysteroid content below the limit of quantification. ^*^Abbreviations: LOC LUS = Locustella luscinioides, TUR MER = Turdus merula, ACR SCH = Acrocephalus schoenobaenus, LUS MEG = Luscinia megarhynchos, ERI RUB = Erithacus rubecula, PRU MOD = Prunella modularis, ACR SCI = Acrocephalus scirpaceus.

**Table 2:** Calibration data for each standard ecdysteroid.

Compound	Regression equation	R^2^	Linear range (ng/mL)	LOD^a^ (ng/mL)	LOQ^b^ (ng/mL)
20E	y = 59.27x + 44.0	0.9988	1.40–1428.6	0.24	0.79
pB	y = 36.61x + 19.1	0.9998	1.40–1428.6	0.20	0.66
pS	y = 78.44x + 28.3	0.9998	1.40–1428.6	0.34	1.12
E	y = 67.86x + 22.0	0.9995	5.58–1428.6	0.77	2.56
2d20E	y = 98.14x + 66.2	0.9996	1.40–1428.6	0.27	0.89
ajC	y = 32.42x–47.7	0.9999	1.40–1428.6	0.38	1.27
Ds	y = 134.57x + 26.8	0.9995	0.35–1428.6	0.14	0.47

Abbreviations: 20-hydroxyecdysone (20E), polypodine B (pB), poststerone (pS), ecdysone (E), 2-deoxy-20-hydroxyecdysone (2d20E), ajugasterone C (ajC), dacryhainansterone (Ds). ^a^LOD: limit of detection.

^b^LOQ: limit of quantification.

**Table 3:** Optimized LC-MS/MS conditions for each standard ecdysteroid.

Compound	Retention time (min)	Quantitative MRM transition	CE (eV)	Qualitative MRM transition	CE (eV)	Fragmentor voltage (V)
20E	2.43	481 > 445	16	481 > 165	24	135
pB	2.53	497 > 443	20	497 > 369	24	135
pS	3.7	363 > 345	12	363 > 215	22	100
E	4.82	447 > 429	20	447 > 109	28	135
2d20E	5.52	465 > 429	16	465 > 355	20	135
ajC	5.65	481 > 427	16	481 > 299	22	135
Ds	6.77	463 > 299	20	463 > 209	26	135

Abbreviations of compounds can be found in the legend of Table 2.
